# Association of visceral adiposity index with incident nephropathy and retinopathy: a cohort study in the diabetic population

**DOI:** 10.1186/s12933-022-01464-1

**Published:** 2022-02-24

**Authors:** Zhiyuan Wu, Siqi Yu, Xiaoping Kang, Yue Liu, Zongkai Xu, Zhiwei Li, Jinqi Wang, Xinlei Miao, Xiangtong Liu, Xia Li, Jingbo Zhang, Wei Wang, Lixin Tao, Xiuhua Guo

**Affiliations:** 1 Beijing Municipal Key Laboratory of Clinical Epidemiology, Department of Epidemiology and Health Statistics, School of Public Health, Capital Medical University, No.10 Xitoutiao, Youanmen Street, Beijing, 100069 China; 2 Beijing Xiaotangshan Hospital, Beijing, China; 3 Department of Mathematics and Statistics, La Trobe University, Melbourne, Australia; 4 Beijing Physical Examination Center, Beijing, China; 5 Centre for Precision Health, Edith Cowan University, Perth, Australia

**Keywords:** Visceral adiposity index, Abdominal obesity, Diabetic nephropathy, Diabetic retinopathy

## Abstract

**Background:**

The association between visceral adiposity index (VAI) and diabetic complications has been reported in cross-sectional studies, while the effect of VAI on complication development remains unclear. This study aims to evaluate the longitudinal association of VAI and Chinese VAI (CVAI) with the incidence of diabetic nephropathy and retinopathy using a Chinese cohort.

**Methods:**

A total of 8 948 participants with type 2 diabetes from Beijing Health Management Cohort were enrolled during 2013–2014, and followed until December 31, 2019. Nephropathy was confirmed by urine albumin/creatinine ratio and estimated glomerular filtration rate; retinopathy was diagnosed using fundus photograph.

**Results:**

The mean (SD) age was 53.35 (14.66) years, and 6 154 (68.8%) were men. During a median follow-up of 4.82 years, 467 participants developed nephropathy and 90 participants developed retinopathy. One-SD increase in VAI and CVAI levels were significantly associated with an increased risk of nephropathy, and the adjusted hazard ratios (HR) were 1.127 (95% CI 1.050–1.210) and 1.165 (95% CI 1.003–1.353), respectively. On contrary, VAI and CVAI level were not associated with retinopathy after adjusting confounding factors.

**Conclusion:**

VAI and CVAI are independently associated with the development of nephropathy, but not retinopathy in Chinese adults with diabetes.

**Supplementary Information:**

The online version contains supplementary material available at 10.1186/s12933-022-01464-1.

## Background

Type 2 diabetes accounts for around 90% of diabetes, becoming a serious public health issue worldwide. It is estimated that 366 million people are expected to have diabetes by 2030, and the vascular complications have become the leading cause of death in type 2 diabetes [1, 2]. The vascular complications severely impact the patients’ life quality and pose a heavy economic burden on the health care system [3].

Abdominal obesity is recognized as one of the important risk factors of cardiometabolic diseases [4], diabetes [5], and diabetic complications [6]. Interestingly, several studies reported that the distribution of adipose tissue rather than the total amount is more crucial in the development of vascular complications [7]. X-ray computed tomography (CT) and magnetic resonance imaging (MRI) are precise methods to detect abdominal adiposity [8]. However, these methods are expensive, time-consuming and require radiation exposure, and thus they are not practical for frequent clinical use and epidemiological research in general population. Waist circumference (WC) is a major clinical indicator of increased visceral fat, which is unable to distinguish between subcutaneous and visceral fat mass, given the different role of subcutaneous and visceral fat [9]. Thus, some surrogate indexes of visceral adiposity have been established, including visceral adiposity index (VAI) and Chinese visceral adiposity index (CVAI) [10, 11]. Previous studies [11, 12] have found that VAI and CVAI could predict the risk of diabetes in adults. Nevertheless, the current evidence about the association between VAI and diabetic complication is limited. A recent study [13] reported the cross-sectional associations of VAI and CVAI with diabetic vascular complications. However, the effect of VAI and CVAI on the development of diabetic complications remains unclear.

Therefore, this study aims to investigate the longitudinal association of VAI and CVAI with the incidence of diabetic nephropathy and retinopathy using a population-based cohort.

## Methods

### Data source and study population

The Beijing health management cohort (BHMC) is an open cohort study conducted in Beijing Xiaotangshan Hospital and Beijing Physical Examination Center in Beijing, China. Briefly, the BHMC study is designed to investigate the risk factors and biomarkers for cardiometabolic diseases, such as metabolic syndrome and type 2 diabetes. The details of the study design have been described in previous studies [14, 15].

**Fig. 1:**
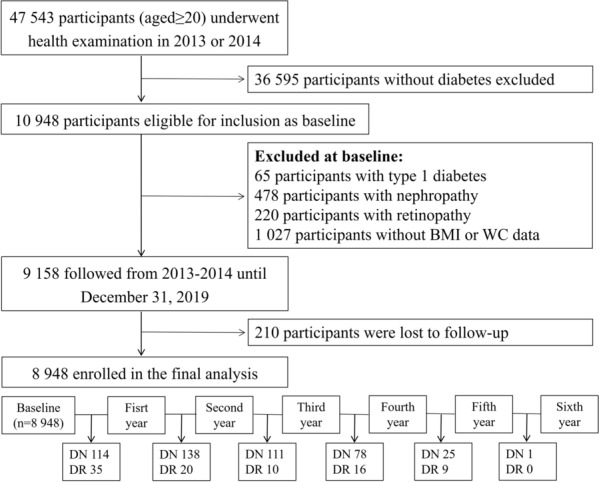
Flow chart of this current study

From 2013 to 2014, a total of 47,543 participants aged 20 years or above underwent a comprehensive health examination, and 10,948 were confirmed to have type 2 diabetes. To minimize the possible influence of reverse causality, 220 participants with a history of retinopathy and 478 participants with nephropathy at baseline were excluded. In addition, we excluded 65 participants with type 1 diabetes and 1027 participants lacking anthropometric measurement data at baseline. Finally, 8948 participants with complete data were annually followed under same identical conditions for the development of nephropathy and retinopathy until December 31, 2019, and the incident cases in each follow-up year were shown in Fig. 1. This study was approved by the Ethics Committee of Capital Medical University (Grant Number: 2020SY031). All participants provided written informed consent.

### Data collection

Baseline data on demographic variables and health information, including age, sex, education level, physical activity, smoking status, drinking status, diagnosis history of diseases and medication use were collected via a standard questionnaire by our trained staff. Educational level was categorized as illiteracy or primary school, middle school, high school or above. Smoking status was defined as ‘current smoker’, ‘former smoker’ and ‘never smoked’. Drinking status was defined as ‘current drinking’ and ‘no current drinking’. Physical activity was defined as having moderate or intense exercise ‘ ≥ 20 min per time and ≥ 4 times per week’. Self-reported health conditions included the physician-diagnosed history of diabetes, hypertension and dyslipidemia. The uses of antidiabetic, antihypertensive and lipid lowering medication were defined as a positive response to the following question: ‘Do you take antidiabetic, antihypertensive or lipid lowering medication regularly following the physician's prescription?’.

The clinical characteristics and biochemical examination data were obtained from the electronic medical record system. Anthropometric measurements include height, weight, and WC. Body mass index (BMI) is calculated as weight (in kilograms)/height^2^ (in metres squared). Systolic blood pressure (SBP) and diastolic blood pressure (DBP) were calculated as the average of two measurements on the right arm using a sphygmomanometer after resting for at least 10 min. The overnight fasting venous blood samples were obtained from all participants, and measured in the central laboratory of Beijing Xiaotangshan Examination Center or Beijing Physical Examination Center. The data of fasting blood glucose, glycated haemoglobin (HbA1c), serum total cholesterol, triglycerides, high-density lipoprotein (HDL) cholesterol, low-density lipoprotein (LDL) cholesterol, serum creatinine, urinary albumin/creatinine ratio (ACR) were collected in this current study. The estimated glomerular filtration rate (eGFR) was calculated using the Chronic Kidney Disease Epidemiology Collaboration serum creatinine equation [16]. Hypertension was defined as SBP ≥ 140 mmHg or DBP ≥ 90 mmHg or use of any antihypertensive medication or self-reported history of hypertension according to the JNC-7 criteria [17]. Diabetes was defined as fasting glucose ≥ 7.0 mmol/l, HbA1c ≥ 6.5% or the use of any antidiabetic medication or self-reported history of diabetes based on the American Diabetes Association [18]. According to the Guidelines on Prevention and Treatment of Dyslipidaemia for Chinese Adults, dyslipidaemia was defined as triglycerides ≥ 2.3 mmol/l, total cholesterol ≥ 6.2 mmol/l, LDL cholesterol ≥ 4.1 mmol/l, HDL cholesterol < 1.0 mmol/l, or any lipid-lowering medication or self-reported history of dyslipidaemia [19].

### Definition of exposure and outcome

Baseline VAI level is calculated using WC, BMI, triglycerides and HDL. CVAI level is calculated using age, WC, BMI, triglycerides and HDL cholesterol. The sex specific formula is as follows:

Men:

VAI = WC/(39.68 + 1.88*BMI)*(triglycerides/1.03)*(1.31/HDL cholesterol).

CVAI = − 267.93 + 0.68*age (years) + 0.03*BMI (kg/m2) + 4.00*WC (cm) + 22.00*lg [triglycerides (mmol/l)]−16.32*HDL cholesterol (mmol/l).

Women:

VAI = WC/(36.58 + 1.89*BMI)*(triglycerides/0.813)*(1.52/HDL cholesterol).

CVAI = − 187.32 + 1.71*age (years) + 4.32*BMI (kg/m2) + 1.12*WC (cm) + 39.76*lg(triglycerides (mmol/l))−11.66*HDL cholesterol (mmol/l).

The VAI and CVAI showed significant correlations with visceral adipose tissue volume as described previously [10, 20].

Diabetic retinopathy was confirmed by ophthalmologists using the 45° four-field stereoscopic digital photography (Carl Zeiss Fundus Camera, Germany) centered at the central point of macula and optic disc, including temporal upper, temporal lower, nasal lower, and nasal upper fields. Non-mydriatic digital images of the retina for both eyes were taken, and the retinopathy was diagnosed according to the International Clinical Diabetic Retinopathy Disease Severity Scale [21]. The retinopathy diagnosis could be divided into four severity: mild non-proliferative; moderate non-proliferative; severe non-proliferative and proliferative. The presence of any severity lesion mentioned above was defined as diabetic retinopathy. Diabetic nephropathy was confirmed as urinary ACR ≥ 30 mg/mmol or eGFR < 60 mL/min/1.73m^2^ according to the organization Kidney Disease Improving Global Outcomes (KDIGO) [22–24].

### Statistical analysis

Categorical variables were presented as number (proportions), and continuous variables were presented as mean (standard deviation, SD) or median (interquartile range, IQR). Difference between groups were compared using Chi-square test for categorical variables, and Student's t-test or Mann–Whitney U test for continuous variables, as appropriate.

Baseline VAI and CVAI were centered and standardized before analysis. The follow-up time was calculated from baseline to the year of nephropathy or retinopathy diagnosis, loss to follow-up, or the end date (December 31, 2019), whichever came first. The Cox proportional hazards models were used to investigate the associations of VAI and CVAI with the risk of incident nephropathy and retinopathy. To adjust for potential confounding factors, the following models were established: Model 1, adjusted for age and sex; Model 2, adjusted for age, sex, education level, smoking status, drinking status, physical activity, BMI group, hypertension, dyslipidaemia, fasting glucose and the use of antidiabetic medication. The hazard ratio (HR) and 95% confidence interval (CI) were provided. The restricted cubic spline function was used to analyze the dose–response relationship of VAI and CVAI with outcome. The discriminatory power of VAI and CVAI was shown using time-dependent receiver operating characteristics (ROC) curve and C-statistic. To identify the modification effect of potential variables, the interactive terms were tested in the model. In addition, we calculated the associations of the tertiles of VAI and CVAI with the diabetic complications, and the individuals were divided into lower, middle and upper groups of VAI and CVAI.

We performed multiple sensitivity analyses. First, participants experiencing a event of nephropathy or retinopathy within one year were excluded. Second, we repeated the analysis in people without the use of antidiabetic medication. Third, HbA1c level was further adjusted to explore the stability of our findings.

All the analyses mentioned above were conducted using R software, version 4.1.0 (R Foundation). The difference was considered statistically significant at two-side significance level of P < 0.05.

## Results

The final analysis included 8 948 individuals with type 2 diabetes, and 6 154 (68.8%) were men. The mean (SD) age of this population was 53.35 (14.66) years. The median [IQR] of baseline VAI and CVAI were 1.46 [0.90,2.37] and 112.60 [77.19,143.17]. During a median follow-up of 4.82 years, 467 participants developed nephropathy and 90 participants developed retinopathy. Table 1 shows the baseline characteristics of participants according to the complication development or not. The CVAI levels were significantly higher in the nephropathy and retinopathy groups, while the VAI level was only significantly higher in the nephropathy group. The baseline characteristics of participants according to the tertiles of baseline VAI and CVAI index are shown in Additional file 1: Table S1 and Table S2.

**Table 1:** Baseline characteristics of the study population

	Overall (n = 8948)	DN- (n = 8481)	DN + (n = 467)	P value	DR- (n = 8858)	DR + (n = 90)	P value
Age, years	53.35 (14.66)	52.42 (14.19)	70.27 (12.70)	< 0.001	53.33 (14.70)	55.88 (10.25)	0.101
Men, n (%)	6154 (68.8)	5825 (68.7)	329 (70.4)	0.453	6074 (68.6)	80 (88.9)	< 0.001
Education, n (%)							
Primary school or below	955 (10.7)	900 (10.6)	55 (11.8)	0.320	939 (10.6)	16 (17.8)	0.003
Middle school	6054 (67.7)	5731 (67.6)	323 (69.2)		5988 (67.6)	66 (73.3)	
High school or above	1939 (21.7)	1850 (21.8)	89 (19.1)		1931 (21.8)	8 (8.9)	
Physical activity (n, %)	3755 (42.0)	3546 (41.8)	209 (44.8)	0.228	3721 (42.0)	34 (37.8)	0.483
Current smoking (n, %)	2709 (30.3)	2592 (30.6)	117 (25.1)	0.013	2668 (30.1)	41 (45.6)	0.002
Current drinking (n, %)	5067 (56.6)	4832 (57.0)	235 (50.3)	0.005	5013 (56.6)	54 (60.0)	0.588
BMI, kg/m2	25.48 (3.36)	25.46 (3.37)	25.86 (3.27)	0.012	25.47 (3.37)	26.23 (3.15)	0.033
WC, cm	87.32 (10.18)	87.17 (10.18)	90.02 (9.75)	< 0.001	87.27 (10.18)	91.70 (8.33)	< 0.001
Hypertension (n, %)	3607 (40.3)	3262 (38.5)	345 (73.9)	< 0.001	3559 (40.2)	48 (53.3)	0.015
Dyslipidaemia (n, %)	3328 (37.2)	3138 (37.0)	190 (40.7)	0.120	3282 (37.1)	46 (51.1)	0.008
Antidiabetic (n, %)	1198 (13.4)	1075 (12.7)	123 (26.3)	< 0.001	1128 (12.7)	70 (77.8)	< 0.001
Antihypertensive (n, %)	732 (8.2)	659 (7.8)	73 (15.6)	< 0.001	728 (8.2)	4 (4.4)	0.268
Lipid lowering (n, %)	492 (5.5)	438 (5.2)	54 (11.6)	< 0.001	488 (5.5)	4 (4.4)	0.835
Fasting glucose, mmol/L	5.31 [4.94,5.94]	5.30 [4.93,5.93]	5.50 [5.04,6.32]	< 0.001	5.30 [4.93,5.91]	8.32 [7.21,12.07]	< 0.001
HbA1c, %	5.57 [5.31,5.96]	5.56 [5.30,5.94]	5.80 [5.54,6.24]	< 0.001	5.57 [5.31,5.95]	7.53 [6.54,9.09]	< 0.001
Triglycerides, mmol/L	1.27 [0.89,1.87]	1.27 [0.89,1.88]	1.21 [0.91,1.77]	0.479	1.27 [0.89,1.87]	1.38 [0.96,2.19]	0.143
HDL-C, mmol/L	1.26 [1.06,1.53]	1.26 [1.06,1.53]	1.25 [1.05,1.56]	0.945	1.26 [1.06,1.53]	1.17 [0.97,1.34]	< 0.001
VAI	1.46 [0.90,2.37]	1.45 [0.90,2.36]	1.76 [1.03,2.80]	0.043	1.46 [0.89,2.37]	1.45 [0.95,2.32]	0.984
CVAI	112.60 [77.19,143.17]	111.07 [75.68,141.98]	136.62 [107.49,160.81]	< 0.001	112.44 [76.88,143.07]	125.61 [104.48,154.95]	< 0.001

Data are the mean (SD), median [IQR] or number (%)

To convert fasting glucose to mg/dL, multiply by 18; triglycerides to mg/dL multiply by 28.25

*DR* diabetic retinopathy, *DN* diabetic nephropathy, *BMI* body mass index, *HbA1c* glycated haemoglobin, *HDL-C* high-density lipoprotein cholesterol, *VAI* visceral adiposity index, *CVAI* Chinese visceral adiposity index

**Fig. 2:**
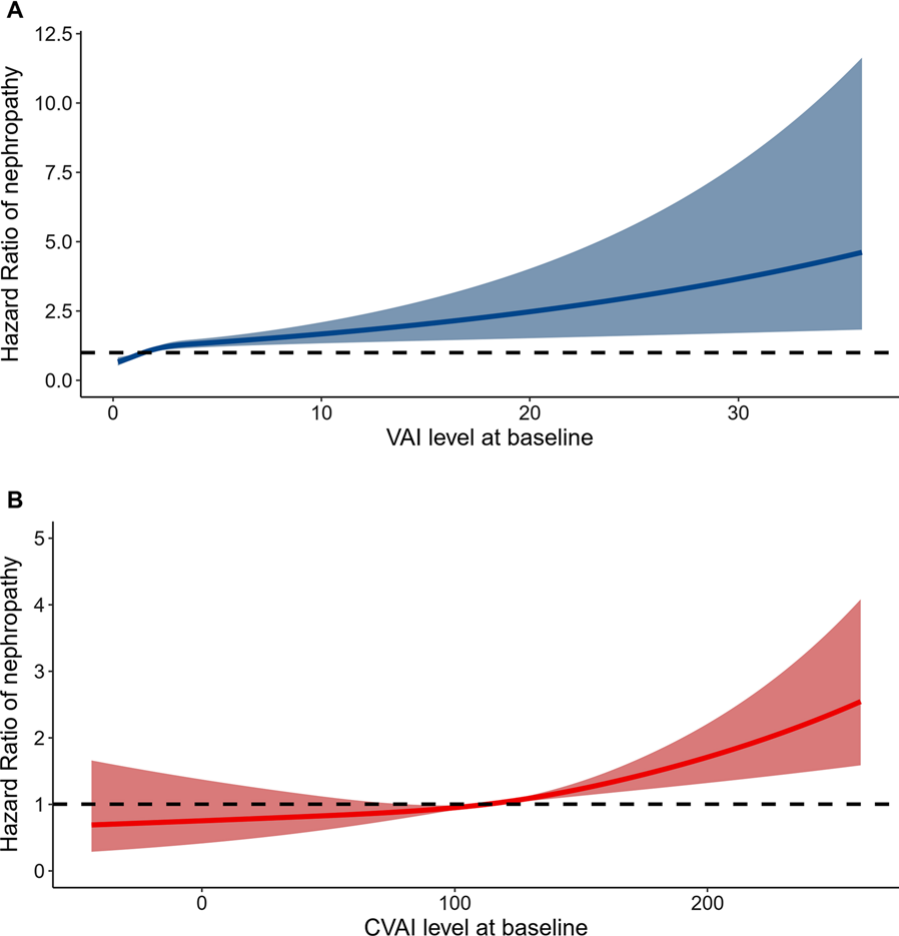
Dose–response relationship of baseline VAI and CVAI with incident nephropathy after adjusting for age and sex

**Fig. 3:**
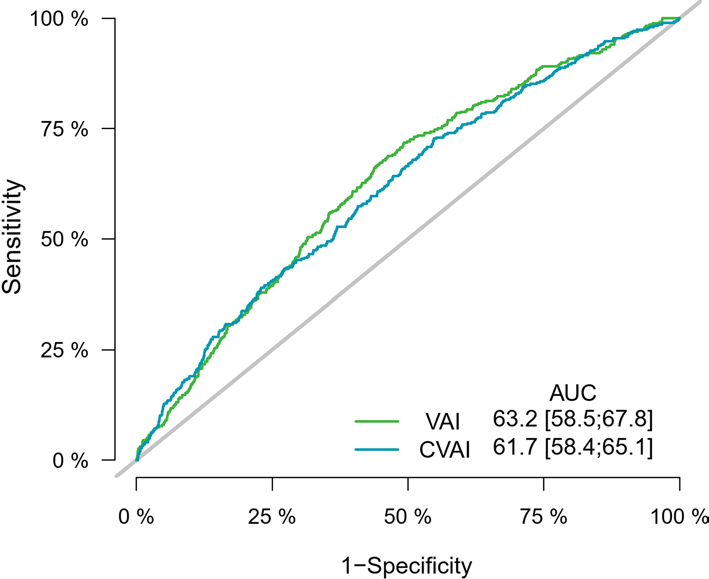
Time-dependent ROC curves of VAI and CVAI for predicting diabetic nephropathy development

In the fully adjusted model, one-SD increase in VAI and CVAI levels were significantly associated with an increased risk of nephropathy, and the HR values were 1.127 (95% CI 1.050–1.210) and 1.165 (95% CI 1.003–1.353), respectively. On contrary, VAI and CVAI levels were not significantly associated with retinopathy after adjusting confounding factors, and the HR values were 1.071 (95% CI 0.950–1.207) and 0.878 (95% CI 0.615–1.252), as shown in Table 2. The full regression results are shown in Additional file 1: Table S3, and fasting glucose level and hypertension had a significant impact on the microvascular complications. The dose–response relationship of VAI and CVAI with the risk of nephropathy development is presented in Fig. 2. The C-statistic values of VAI and CVAI for predicting the development of nephropathy were 0.632 [95% CI 0.585–0.678] and 0.617 [95% CI 0.584–0.651], as shown in Fig. 3. The C-statistic values for predicting retinopathy is presented in Additional file 2: Fig. S1.

**Table 2:** Association of visceral adiposity indexes with the development of diabetic nephropathy and retinopathy

	Hazard ratio (95% CI)			
	Model 1	P value	Model 2	P value
Nephropathy				
VAI per-SD increase	1.154 (1.095–1.217)	< 0.001	1.127 (1.050–1.210)	0.001
CVAI per-SD increase	1.296 (1.159–1.450)	< 0.001	1.165 (1.003–1.353)	0.045
Retinopathy				
VAI per-SD increase	0.739 (0.549–0.995)	0.047	1.071 (0.950–1.207)	0.264
CVAI per-SD increase	1.330 (1.038–1.704)	0.024	0.878 (0.615–1.252)	0.471

Model 1: adjusted for age and sex

Model 2: age, sex, BMI group, education level, smoking status, drinking status, physical activity, hypertension, dyslipidaemia, fasting glucose and use of antidiabetic medication

*VAI* visceral adiposity index, *CVAI* Chinese visceral adiposity index

In addition, we observed no significant interaction effect of age, sex, obesity, smoking with VAI and CVAI for incident nephropathy (Additional file 1: Table S4). Table 3 summarizes the association of the tertiles of VAI and CVAI with the incidence of nephropathy and retinopathy. Individuals in the highest tertile of VAI were significantly associated with an increased risk of nephropathy, compared with those in the lowest tertile (adjusted HR: 1.338, 95% CI 1.033–1.735, P value: 0.028). Significant association was not observed between CVAI tertiles and incident nephropathy. Tertiles of VAI and CVAI were not associated with the development of retinopathy.

**Table 3:** Association of the tertiles of visceral adiposity indexes with the development of diabetic nephropathy and retinopathy

	Hazard ratio (95% CI)			
	Model 1	P value	Model 2	P value
Nephropathy				
VAI (ref: lower)	–		–	
Middle	1.222 (0.976–1.531)	0.080	1.098 (0.873–1.382)	0.425
Upper	1.571 (1.251–1.973)	< 0.001	1.338 (1.033–1.735)	0.028
CVAI (ref: lower)	–		–	
Middle	1.136 (0.851–1.516)	0.387	0.989 (0.737–1.325)	0.938
Upper	1.690 (1.293–2.208)	< 0.001	1.317 (0.975–1.780)	0.073
Retinopathy				
VAI (ref: lower)	–		–	
Middle	1.025 (0.587–1.790)	0.930	0.765 (0.431–1.361)	0.363
Upper	1.536 (0.915–2.578)	0.104	0.696 (0.354–1.367)	0.292
CVAI (ref: lower)	–		–	
Middle	2.198 (1.119–4.317)	0.022	1.369 (0.688–2.722)	0.371
Upper	2.335 (1.188–4.589)	0.014	1.134 (0.518–2.484)	0.754

Model 1: adjusted for age and sex

Model 2: age, sex, BMI group, education level, smoking status, drinking status, physical activity, hypertension, dyslipidaemia, fasting glucose and use of antidiabetic medication;

*VAI* visceral adiposity index, *CVAI* Chinese visceral adiposity index

**Fig. 4:**
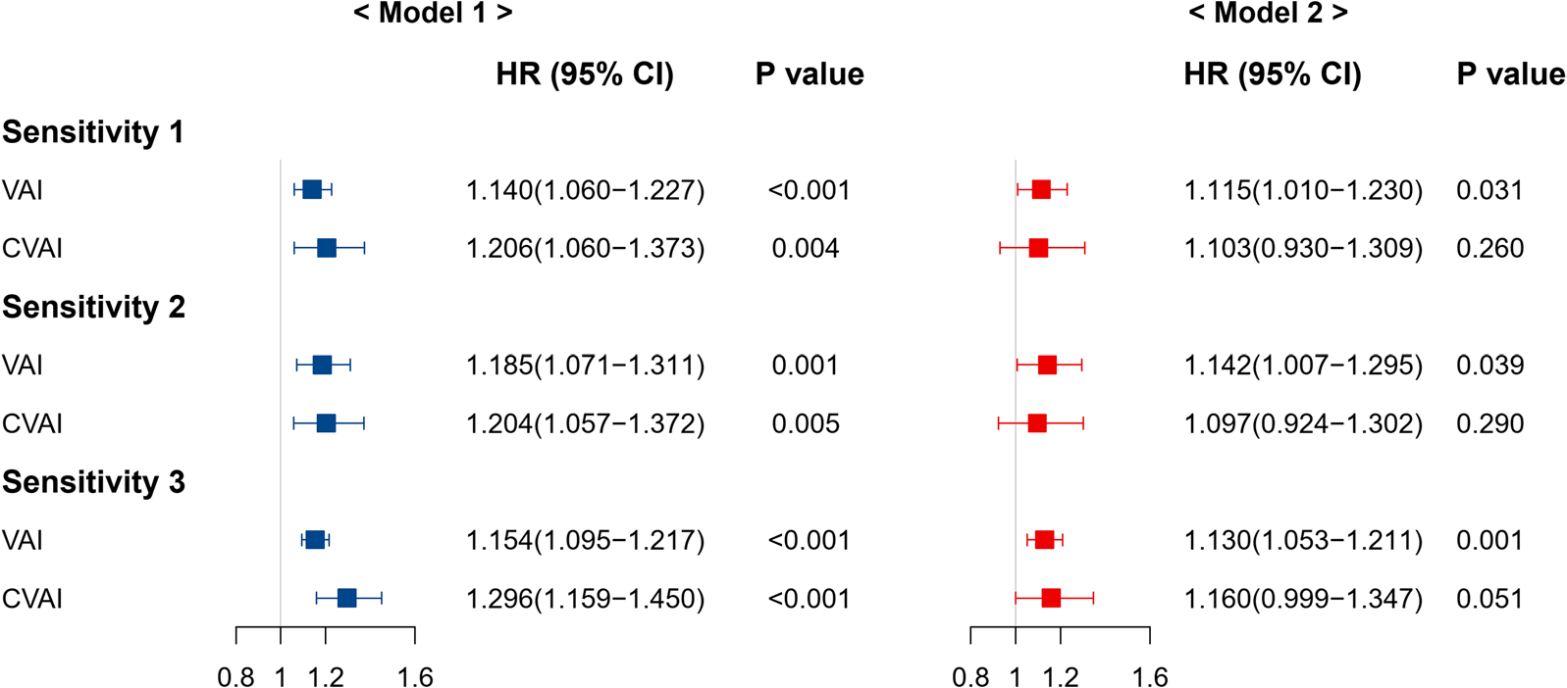
Association of VAI and CVAI with incident nephropathy in sensitivity analyses

We repeated the analysis after excluding participants who developed nephropathy within one year. Only VAI was significantly associated with incident nephropathy (HR: 1.115, 95% CI 1.010–1.230). After excluding participants using antidiabetic medication, we found that VAI, not CVAI was significantly associated with incident nephropathy (HR: 1.142, 95% CI 1.007–1.295). The results remained almost consistent when HbA1c level was further adjusted, and VAI was independently associated with nephropathy development (HR: 1.130, 95% CI 1.053–1.211), as shown in Fig. 4.

## Discussion

This study evaluated the longitudinal association of visceral adiposity index with the development of nephropathy and retinopathy in a diabetic population. In the current analysis, we found that One-SD increase of VAI and CVAI levels were significantly associated with an increased risk of nephropathy, but not retinopathy in Chinese adults with diabetes after adjusting confounding factors. In the multiple sensitivity analyses, we found that VAI was more independently associated with incident nephropathy than CVAI.

Previous studies found that excess visceral fat, but not general adiposity, is strongly correlated with incident prediabetes and type 2 diabetes in obese adults [25, 26]. Some studies indicated that, as a simple indicator of visceral adipose function, VAI and CVAI could increase the risk of prediabetes and type 2 diabetes. Xia et al. [11, 27] reported that CVAI is a stronger predictor of prediabetes and diabetes than traditional indexes such as BMI and WC. Similarly, TZou et al. [28] found that CVAI and VAI may serve as potential indicators of diabetes. In addition, some studies reported that VAI is correlated with the risk of macrovascular complications in diabetic population [29, 30]. However, data on the associations of VAI and CVAI with diabetic microvascular complications are relatively limited. Several cross-sectional studies reported that VAI is an independent risk factor for excess urinary albumin excretion and diabetic nephropathy [31–33], and CVAI is closely associated with the prevalence of cardiovascular diseases and diabetic nephropathy [13]. In this current study, we found that VAI and CVAI levels at baseline are positively associated with the development of nephropathy in diabetic population using a cohort design.

Although the underlying mechanisms of VAI with diabetic nephropathy have not been fully illustrated, there are several possible explanations accounting for the observed associations. First, VAI is a representative marker to assess visceral adiposity which is strongly related with insulin resistance [34], and insulin resistance may lead to hypo-inflammation [35], endothelium dysfunction [36], and oxidative stress [37], contributing to the development of microangiopathy. Podocytes are insulin sensitive renal cells, and thus the insulin resistance is more likely to cause kidney damage [38]. Second, VAI is a valuable indicator of visceral adipose function [10]. The release of free fatty acids from central and visceral adipose tissue can increase the secretion of pro-inflammatory factors [39], such as TNF-α and interleukin-6, which could cause glomerular endothelial dysfunction and increase urine albumin. Third, obesity may also directly affect renal pathophysiology through the altered renal hemodynamics, as well as the production of adipokines and growth factors [40]. Adipokines are involved in microvascular injury and kidney damage by mediating endothelial dysfunction, inducing oxidative stress, inflammation, activation of the renin–angiotensin–aldosterone system [41] and endoplasmic reticulum stress [42]. All these mechanisms could partly explain the positive association between VAI and the development of nephropathy.

Wan et al. [13] found that VAI and CVAI were associated with a higher prevalence of diabetic nephropathy. While multiple sensitivity analyses in our study did not find a stable association between baseline CVAI and the incidence of diabetic nephropathy. The distinct formulas of calculating VAI and CVAI may account for this difference. In our study, the individuals with diabetic nephropathy were much older. Previous studies have shown that visceral adipose tissue is not associated with incident atherosclerotic cardiovascular events in older men [43]. Therefore, visceral adipose tissue may not have a strong adverse effect on health in relatively older people. CVAI is calculated based on age, BMI, WC, triglycerides, and HDL-C, while VAI is calculated independent of age and thus may be more useful for predicting the development of diabetic nephropathy. More studies in Chinese population are needed to confirm the association of VAI and CVAI with incident diabetic complications.

In this study, we did not find a positive association of VAI and CVAI with incident diabetic retinopathy, which is different from some other studies. Moh et al. [44] reported that visceral adiposity is associated with diabetic retinopathy in an Asian cohort with longstanding type 2 diabetes for 10 years. Of note, the cumulative incidence of diabetic retinopathy was only 1.0% in our study, while the annual incidence of diabetic retinopathy ranged from 2.2% to 12.7% from other Asia, North America, Caribbean, and sub-Saharan Africa studies [45], which indicates the participants in our cohort were at a relatively early stage of diabetes. In addition, the participants were annually physically examined and thus advised by our trained staff about the glucose control. The glucose and HbA1c levels were not severely high in this cohort, which could partially explain the low incidence of diabetic retinopathy. In addition, Moh et al. [44] found that the association between visceral adiposity and diabetic retinopathy became insignificant after controlling for ACR and eGFR, and thus the relationship may be attributable to vascular injury reflected by coexisting renal burden. Therefore, the visceral adiposity could be more strongly associated with renal function and nephropathy, which is also indicated in our study.

Our study is based on a population cohort of people with type 2 diabetes, which could explain the longitudinal associations of VAI and CVAI with the development of diabetic complications. In addition, the data were obtained from the central medical system or using the standardized questionnaire, which allow us to adjust for the potential confounding factors. However, several limitations in our study should be acknowledged. First, the visceral adiposity was not directly measured by MRI method, and we failed to examine the consistence between actual visceral fat mass and VAI in Chinese population. Second, the exact duration of diabetes in this cohort was unavailable, and the duration is an important risk factor of diabetic complication, which could bias the relationship between VAI and diabetic nephropathy. Third, information about the neurological complication is unavailable in this current cohort. We failed to assess the longitudinal association between VAI and the neuropathy development in diabetic population.

## Conclusions

Our findings suggest that elevated levels of VAI and CVAI at baseline are significantly associated with increased risk of incident nephropathy, but not retinopathy in diabetic population.

## Supplementary Information

**Additional file 1: Table S1.** Baseline characteristics according to tertiles groups of VAI. **Table S2: **Baseline characteristics according to tertiles groups of CVAI. **Table S3:** Full regression results of VAI and CVAI with diabetic nephropathy and retinopathy. **Table S4: **Interaction effect test of age, sex, obesity and smoking with incident nephropathy.

**Additional file 2: Figure S1.** Time-dependent ROC curves of VAI and CVAI for predicting diabetic retinopathy development.

## Data Availability

The datasets used and/or analysed during the current study are available from the corresponding author (Dr. Xiuhua Guo) on reasonable request.

## Abbreviations

VAI: Visceral adiposity index
CVAI: Chinese visceral adiposity index
BHMC: Beijing Health Management Cohort
WC: Waist circumference
BMI: Body mass index
SBP: Systolic blood pressure
DBP: Diastolic blood pressure
HbA1c: Glycated haemoglobin
HDL: High-density lipoprotein
LDL: Low-density lipoprotein
ACR: Albumin/creatinine ratio
eGFR: Estimated glomerular filtration rate
ROC: Receiver operating characteristics
