# Supplementary material for: Association of visceral adiposity index with incident nephropathy and retinopathy: a cohort study in the diabetic population
Source: Cardiovasc Diabetol. 2022 Feb 24;21:32. doi: 10.1186/s12933-022-01464-1 (PMC8876445; doi:10.1186/s12933-022-01464-1)
Supplement: Supplementary file 1 — Additional file 1: Table S1. Baseline characteristics according to tertiles groups of VAI. Table S2: Baseline characteristics according to tertiles groups of CVAI. Table S3: Full regression results of VAI and CVAI with diabetic nephropathy and retinopathy. Table S4: Interaction effect test of age, sex, obesity and smoking with incident nephropathy. [file 12933_2022_1464_MOESM1_ESM.docx]

**Supplementary files**

**Table S1:** Baseline characteristics according to tertiles groups of VAI.

|  | Lower (n=2953) | Middle (n=2953) | Upper (n=3042) | P value |
| --- | --- | --- | --- | --- |
| Age, years | 54.16(16.90) | 53.95(14.09) | 51.99(12.64) | <0.001 |
| Men, n (%) | 1832(62.0) | 2060(69.8) | 2262(74.4) | <0.001 |
| Education, n (%) |  |  |  | 0.117 |
| Primary school or below | 295(10.0) | 307(10.4) | 353(11.6) |  |
| Middle school | 2022(68.5) | 1974(66.8) | 2058(67.7) |  |
| High school or above | 636(21.5) | 672(22.8) | 631(20.7) |  |
| Physical activity (n, %) | 1278(43.3) | 1250(42.3) | 1227(40.3) | 0.062 |
| Current smoking (n, %) | 668(22.6) | 903(30.6) | 1138(37.4) | <0.001 |
| Current drinking (n, %) | 1493(50.6) | 1691(57.3) | 1883(61.9) | <0.001 |
| BMI, kg/m2 | 23.74(3.13) | 25.72(3.08) | 26.93(3.08) | <0.001 |
| WC, cm | 81.92(10.00) | 88.16(9.29) | 91.73(8.68) | <0.001 |
| Hypertension (n, %) | 947(32.1) | 1242(42.1) | 1418(46.6) | <0.001 |
| Dyslipidaemia (n, %) | 337(11.4) | 763(25.8) | 2228(73.2) | <0.001 |
| Antidiabetic (n, %) | 353(12.0) | 395(13.4) | 450(14.8) | 0.005 |
| Fasting glucose, mmol/L | 5.11[4.80,5.56] | 5.31[4.96,5.86] | 5.55[5.10,6.56] | <0.001 |
| HbA1c, % | 5.48[5.23,5.77] | 5.59[5.34,5.95] | 5.67[5.38,6.21] | <0.001 |
| Triglycerides, mmol/L | 0.78[0.64,0.92] | 1.26[1.09,1.47] | 2.19[1.80,2.96] | <0.001 |
| HDL-C, mmol/L | 1.59[1.39,1.83] | 1.26[1.11,1.43] | 1.03[0.90,1.17] | <0.001 |
| VAI | 0.74[0.56,0.89] | 1.44[1.25,1.67] | 2.93[2.34,4.20] | <0.001 |
| CVAI | 70.73[37.48,103.01] | 111.33[86.18,137.84] | 141.89[119.59,165.08] | <0.001 |

Data are the mean (SD), median [IQR] or number (%).

BMI, body mass index; HbA1c: glycated haemoglobin; HDL-C, high-density lipoprotein cholesterol; VAI, visceral adiposity index; CVAI, Chinese visceral adiposity index.

**Table S2:** Baseline characteristics according to tertiles groups of CVAI.

|  | Lower (n=2953) | Middle (n=2953) | Upper (n=3042) | P value |
| --- | --- | --- | --- | --- |
| Age, years | 46.60(14.05) | 55.04(13.33) | 58.28(14.04) | <0.001 |
| Men, n (%) | 1322(44.8) | 2275(77.0) | 2557(84.1) | <0.001 |
| Education, n (%) |  |  |  | <0.001 |
| Primary school or below | 247(8.4) | 326(11.0) | 382(12.6) |  |
| Middle school | 1960(66.4) | 1991(67.4) | 2103(69.1) |  |
| High school or above | 746(25.3) | 636(21.5) | 557(18.3) |  |
| Physical activity (n, %) | 1170(39.6) | 1342(45.4) | 1243(40.9) | <0.001 |
| Current smoking (n, %) | 622(21.1) | 957(32.4) | 1130(37.1) | <0.001 |
| Current drinking (n, %) | 1437(48.7) | 1744(59.1) | 1886(62.0) | <0.001 |
| BMI, kg/m2 | 22.63(2.36) | 25.41(2.08) | 28.30(2.80) | <0.001 |
| WC, cm | 77.08(6.45) | 87.59(4.64) | 96.98(6.93) | <0.001 |
| Hypertension (n, %) | 454(15.4) | 1232(41.7) | 1921(63.1) | <0.001 |
| Dyslipidaemia (n, %) | 395(13.4) | 1122(38.0) | 1811(59.5) | <0.001 |
| Antidiabetic (n, %) | 168(5.7) | 432(14.6) | 598(19.7) | <0.001 |
| Fasting glucose, mmol/L | 5.01[4.74,5.35] | 5.36[5.02,6.00] | 5.66[5.20,6.79] | <0.001 |
| HbA1c, % | 5.41[5.18,5.63] | 5.60[5.34,5.99] | 5.78[5.48,6.38] | <0.001 |
| Triglycerides, mmol/L | 0.86[0.67,1.13] | 1.35[1.02,1.82] | 1.83[1.31,2.67] | <0.001 |
| HDL-C, mmol/L | 1.53[1.30,1.79] | 1.23[1.07,1.44] | 1.09[0.94,1.28] | <0.001 |
| VAI | 0.85[0.59,1.22] | 1.55[1.07,2.22] | 2.40[1.57,3.76] | <0.001 |
| CVAI | 58.07[32.86,76.78] | 111.97[102.38,121.50] | 154.75[142.59,173.22] | <0.001 |

Data are the mean (SD), median [IQR] or number (%).

BMI, body mass index; HbA1c: glycated haemoglobin; HDL-C, high-density lipoprotein cholesterol; VAI, visceral adiposity index; CVAI, Chinese visceral adiposity index.

**Table S3:** Full regression results of VAI and CVAI with diabetic nephropathy and retinopathy.

|  | Nephropathy | | Retinopathy | |
| --- | --- | --- | --- | --- |
|  | Hazard Ratio (95% CI) | P value | Hazard Ratio (95% CI) | P value |
| VAI (one-SD) | 1.127(1.050-1.210) | 0.001 | 1.071(0.950-1.207) | 0.264 |
| Age | 1.087(1.078-1.096) | <0.001 | 1.010(0.992-1.027) | 0.283 |
| Women | 1.442(1.172-1.776) | 0.001 | 0.479(0.239-0.962) | 0.039 |
| High school or above | 1.163(0.875-1.544) | 0.298 | 0.620(0.355-1.084) | 0.094 |
| Physical activity | 1.144(0.949-1.380) | 0.159 | 0.821(0.530-1.272) | 0.377 |
| Current drinking | 1.205(0.986-1.471) | 0.068 | 0.965(0.612-1.523) | 0.880 |
| Current smoking | 0.988(0.791-1.234) | 0.917 | 1.245(0.796-1.945) | 0.337 |
| Dyslipidaemia | 1.138(0.938-1.380) | 0.190 | 1.219(0.752-1.975) | 0.422 |
| Hypertension | 1.665(1.331-2.082) | <0.001 | 1.005(0.642-1.571) | 0.983 |
| Fasting glucose | 1.052(0.991-1.116) | 0.097 | 1.500(1.428-1.576) | <0.001 |
| Obesity | 1.192(0.957-1.484) | 0.117 | 0.712(0.420-1.208) | 0.208 |
|  |  |  |  |  |
| CVAI (one-SD) | 1.165(1.003-1.353) | 0.045 | 0.878(0.615-1.252) | 0.471 |
| Age | 1.083(1.074-1.092) | <0.001 | 1.016(0.997-1.034) | 0.094 |
| Women | 1.476(1.199-1.817) | <0.001 | 0.450(0.222-0.915) | 0.027 |
| High school or above | 1.154(0.869-1.532) | 0.321 | 0.631(0.360-1.105) | 0.107 |
| Physical activity | 1.147(0.951-1.384) | 0.150 | 0.862(0.557-1.334) | 0.505 |
| Current drinking | 1.192(0.975-1.456) | 0.086 | 0.967(0.614-1.525) | 0.887 |
| Current smoking | 0.984(0.788-1.229) | 0.886 | 1.234(0.790-1.928) | 0.356 |
| Dyslipidaemia | 1.142(0.938-1.390) | 0.187 | 1.020(0.627-1.661) | 0.936 |
| Hypertension | 1.625(1.297-2.034) | <0.001 | 0.986(0.627-1.550) | 0.951 |
| Fasting glucose | 1.052(0.991-1.117) | 0.096 | 1.487(1.413-1.565) | <0.001 |
| Obesity | 1.034(0.793-1.347) | 0.807 | 0.787(0.419-1.480) | 0.457 |

Abbreviations: VAI, visceral adiposity index; CVAI, Chinese visceral adiposity index.

Obesity refers to BMI ≥28.0 kg/m2.

**Table S4:** Interaction effect test of age, sex, obesity and smoking with incident nephropathy.

|  | P for interaction | |
| --- | --- | --- |
|  | VAI | CVAI |
| Age | 0.459 | 0.606 |
| Sex | 0.193 | 0.295 |
| Obesity (BMI ≥ 28.0 kg/m2) | 0.626 | 0.119 |
| Current smoking | 0.435 | 0.646 |
