# Supplementary figures and images for: Association of visceral adiposity index with incident nephropathy and retinopathy: a cohort study in the diabetic population
Source: Cardiovasc Diabetol. 2022 Feb 24;21:32. doi: 10.1186/s12933-022-01464-1 (PMC8876445; doi:10.1186/s12933-022-01464-1)

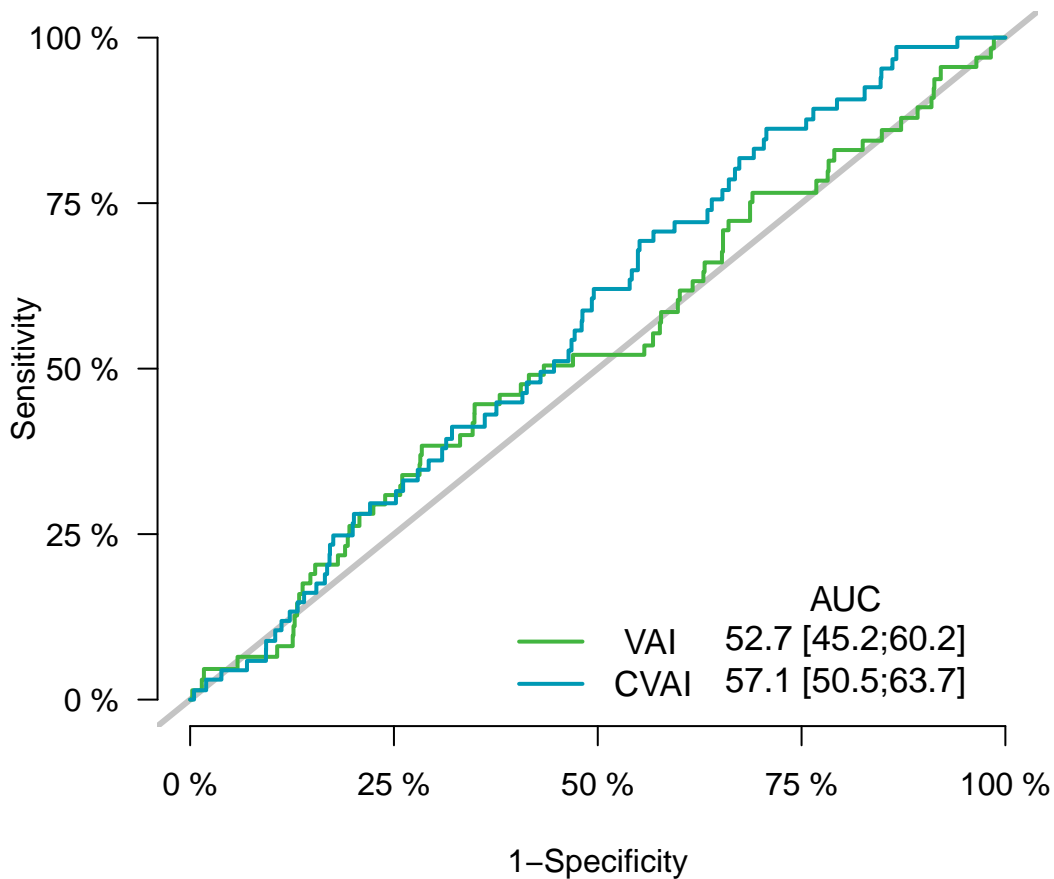

Supplement: Supplementary file 2 — Additional file 2: Figure S1. Time-dependent ROC curves of VAI and CVAI for predicting diabetic retinopathy development. [file 12933_2022_1464_MOESM2_ESM.pdf]
